# JAM3: A prognostic biomarker for bladder cancer via epithelial–mesenchymal transition regulation

**DOI:** 10.17305/bb.2024.9979

**Published:** 2024-08-01

**Authors:** Zhong-qi Pang, Jian-she Wang, Jin-feng Wang, Ya-xuan Wang, Bo Ji, Yi-dan Xu, Jia-xin He, Lu Zhang, Li-qiu Zhang, Bei-chen Ding, Yang Liu, Ming-hua Ren

**Affiliations:** 1 Department of Urology, First Affiliated Hospital of Harbin Medical University, Harbin, Heilongjiang, China; 2 Teaching Experiment Center of Biotechnology, Harbin Medical University, Harbin, Heilongjiang, China; 3 Department of Urology, The Eighth Affiliated Hospital of Sun Yat sen University, Shenzhen, Guangdong, China

**Keywords:** Junctional adhesion molecule 3 (JAM3), bladder cancer (BC), epithelial–mesenchymal transition (EMT), prognostic biomarker, immune cell infiltration

## Abstract

Understanding the intricate relationship between prognosis, immune function, and molecular markers in bladder cancer (BC) demands sophisticated analytical methods. To identify novel biomarkers for predicting prognosis and immune function in BC patients, we combined weighted gene co-expression network analysis (WGCNA) and least absolute shrinkage and selection operator (LASSO) regression analysis. This was conducted using data from The Cancer Genome Atlas (TCGA) and Gene Expression Omnibus (GEO) databases. Ultimately, we screened the junctional adhesion molecule 3 (JAM3) as an independent risk factor in BC. High levels of JAM3 were linked to adverse clinical parameters, such as higher T and N stages. Additionally, a JAM3-based nomogram model accurately predicted 1-, 3- and 5-year survival rates of BC patients, indicating potential clinical utility. Functional enrichment analysis revealed that high JAM3 expression activated the calcium signaling pathway, the extracellular matrix (ECM)-receptor interaction, and the PI3K-Akt signaling pathway, and was positively correlated with genes associated with epithelial–mesenchymal transition (EMT). Subsequently, we found that overexpression of JAM3 promoted the migration and invasion abilities in BC cells, regulating the expression levels of N-Cadherin, matrix metallopeptidase 2 (MMP2), and Claudin-1 thereby promoting EMT levels. Additionally, we showed that JAM3 was negatively correlated with anti-tumor immune cells such as CD8+ T cells, while positively correlated with pro-tumor immune cells such as M2 macrophages, suggesting its involvement in immune cell infiltration. The immune checkpoint CD200 also showed a positive correlation with JAM3. Our findings revealed that elevated JAM3 levels are predictive of poor prognosis and immune cell infiltration in BC patients by regulating the EMT process.

## Introduction

Bladder cancer (BC) is the most commonly diagnosed tumor in the urinary system with high mortality, causing a societal burden worldwide [1, 2]. Unfortunately, the mechanisms underlying BC initiation, proliferation, and progression remain largely unknown. Known risk factors include age, gender, cigarettes, genetic factors, and other environmental influences [3]. At present, the mainstay treatment for BC including surgery, chemotherapy, and immune therapy has markedly improved patient outcomes. However, high recurrence rates, as well as the side effects or the limited effectiveness in advanced or metastatic stages of BC continue to result in significant mortality [4]. Accordingly, it is imperative to discover new methods or biomolecules with effective value on the early detection or improvement of BC prognosis.

Based on whether the tumor cells invade the muscle layer of the bladder wall, BC can be classified into non-muscle-invasive BC (NMIBC) and muscle-invasive BC (MIBC). MIBC exhibits a more aggressive nature, correlates with a higher level of T-stage, and has more significant genetic heterogeneity, in which impaired DNA damage response and repair pathways are very common [5]. Additionally, studies have been done to use changed genes that are strongly linked to the development of BC to classify the disease into several molecular subtypes [6].

Immunotherapy has emerged as a pivotal treatment for advanced and metastatic BC. The behavior of BC cells, their response to treatment, and the prognosis of individuals with BC are all influenced by the immunological microenvironment, which has been the focus of research [7, 8]. Note that the immune checkpoint inhibitors (ICIs) are the first-line treatment among immunotherapies, targeting expression levels of programmed cell death protein 1 (PD-1), programmed death-ligand 1 (PD-L1), and cytotoxic T-lymphocyte-associated protein 4 (CTLA4), which are associated with immunotherapy response [9]. A recent study demonstrated that targeting androgen receptor (AR) to reduce PD-L1 expression can enhance the tumor-killing abilities of NK cells [10]. However, the success rate of anti-PD1/PDL1 treatment in advanced BC patients is estimated to be around 20% [11]. Besides, it is reported that the therapeutic response of ICI treatment was closely associated with tumor microenvironment (TME) and tumor-infiltrating immune cells [12]. Nevertheless, the fact that immune cells can act as protective factors (anti-tumor immunity) or risk factors (pro-tumor immunity) in the TME makes them a “double-edged sword” [13]. For example, the roof plate-specific spondin (RSPO) family has been found to influence the development of BC by regulating the invasion of CD4 T cells and macrophages [14]. Still, investigating the tumor-infiltrating immune cell levels could help understand the mechanism of tumor immunity and predict the ICI responses.

The occurrence of genetic alterations is believed to be closely linked to BC tumorigenesis [15]. Therefore, investigating the genetic alterations might offer opportunities to further understand biological changes in BC. In recent years, bioinformatics has been an important method in cancer research. One widely used approach is analyzing the expression of differentially expressed genes (DEGs) [16]. Another powerful method is the weighted gene co-expression network analysis (WGCNA), which can reveal patterns of gene expression and identify highly significant genes associated with specific traits [17]. Additionally, the least absolute shrinkage and selection operator (LASSO) regression analysis can be utilized to identify the most important combination of independent variables and regression coefficients for the most accurate predictive model [18].

Junctional adhesion molecule 3 (JAM3), located on the 11q25 region of the human chromosome, is a member of the JAM family, which are cell–cell adhesion molecules of the immunoglobulin superfamily. JAM3 is expressed in various tissues and plays a crucial role in cell junctions, cell polarity, and motility [19–21]. It has been proposed that JAM3 participates in leukocyte–platelet interactions, as well as angiogenesis and brain development [22, 23]. In the field of cancer research, the expression of JAM3 is silenced by the gene methylation in colorectal cancer and esophageal cancer, showing a close relationship between gene functions of JAM3 and its relative methylation level [24, 25]. It is important to note that the roles of JAM3 are reported different and controversial in multiple cancers. In leukemia, JAM3 maintains leukemia-initiating cell function through the LRP5/AKT/β-catenin/CCND1 signaling pathway and is associated with poor prognosis in leukemia [26]. The methylation level of JAM3 is identified as an independent risk factor in esophageal cancer by activating the Wingless-related integration site (Wnt) signaling pathway, showing the tumor suppression function of JAM3 [25]. However, the roles of JAM3 in BC are largely unknown at present.

The epithelial–mesenchymal transition (EMT) was considered as a classic molecular mechanism of tumor metastasis, with remarkable changes in expression levels of several crucial EMT-related proteins, including zinc finger E-box binding homeobox (ZEBs) proteins, Snail proteins, matrix metalloproteinases (MMPs) proteins, Claudin-1, Vimentin, Cadherin-1 (CDH1), and Cadherin-2 (CDH2) which gained a lot of attention on the treatment for multiple cancers [27]. While the involvement of JAM3 in EMT has been documented in gastric cancer [28], its regulation in BC still lacks sufficient evidence.

In summary, we obtained 16 genes by combining DEGs, WGCNA, and applied LASSO regression analysis. The results of univariate and multivariate cox analysis of these 16 genes revealed that only JAM3 was an independent prognostic factor in BC. Previous studies have not been able to elucidate the specific role of JAM3 in BC. Therefore, further research on the relationship between JAM3 and BC cell behavior, particularly in relation to EMT, is necessary for a comprehensive understanding of its function in this disease. This understanding can contribute to prognostication, tumor progression, and guide treatment strategies to improve patient survival. Our findings indicate that high levels of JAM3 are a significant prognostic indicator for predicting unfavorable outcomes, adverse clinical features, and reduced immune cell infiltration in BC.

In this study, we identified JAM3 as an independent risk factor in BC, with further investigation to assess its prognostic value and perform preliminary functional exploration to indicate that JAM3 regulates the EMT process in BC, thus providing a new marker for predicting the prognosis and immune functions of BC patients.

## Materials and methods

### Data download and collation

In this study, we acquired transcriptome data and clinical information for 394 BC cases and 87 normal cases from six Gene Expression Omnibus (GEO) cohorts (GSE3167, GSE13507, GSE52519, GSE65635, GSE100926, and GSE120736) available on the GEO database (http://www.ncbi.nlm.nih.gov/geo/). The clinical traits of the samples are displayed in Figure S4. Meanwhile, transcriptome data, related clinical information, and DNA methylation data were downloaded from The Cancer Genome Atlas (TCGA) database (https://tcga-data.nci.nih.gov/tcga/) for 431 cases (412 BC cases and 19 normal cases). Then, we used R package sva (version 3.44.0) to batch the correction of these six GEO cohorts and obtained a vast GEO cohort for further investigation.

### Identification of BC-related genes in the vast Gene Expression Omnibus (GEO) cohort

Firstly, we standardized all transcriptomics data from the vast GEO cohort by applying log2(*x* + 1). Then, we applied the edge R package with FDR <0.05 and |log2FC| ≥1 to identify DEGs of transcriptomics in both TCGA and GEO cohorts. Meanwhile, we used the WGCNA for identifying BC-related genes preliminarily by applying the R package WGCNA (version 1.71). During the WGCNA analysis, we set the power value as seven to complete the process and set Module Membership to 0.8 and Gene significance to 0.2 to identify BC-related genes in the most significant module. After that, we intersected DEGs with BC-related genes and applied the LASSO cox analysis to further filtrate these genes for identifying BC-related genes.

### Identification of prognostic BC-related genes

To identify those BC-related genes with prognostic value for further investigation, we applied univariate and multivariate Cox regression analyses and Kaplan–Meier analysis by using data from the TCGA cohort.

### Gene biological function and immune function analysis

We applied Gene Set Variation Analysis (GSVA), Disease Ontology (DO), Gene Ontology (GO), Kyoto Encyclopedia of Genes and Genomes (KEGG), and Gene Set Enrichment Analysis (GSEA) for gene functional or pathway enrichment analysis. Then, R package limma (version 3.52.4) and algorithm of cibersort were used to assess the immune infiltration levels of BC cases.

### Protein–protein interaction (PPI) network

We generated a PPI network for correlated expressed genes by using STRING (https://string-db.org/) and Cytoscape software.

### Cell culture

In this study, we obtained human normal bladder cell line SVHUC-1, and human BC cell lines T24, RT112, and UMUC3 from the Institute of Biochemistry and Cell Biology, Chinese Academy of Sciences (Shanghai, China). The SVHUC, T24, and RT112 cells were cultured in the RPMI-1640 (Gibco) supplemented with 10% fetal bovine serum and 1% penicillin/streptomycin, while UMUC3 cell was cultured in the DMEM (Gibco) supplemented with 10% fetal bovine serum and 1% penicillin/streptomycin. Both cell lines were cultured at 37 ^∘^C and in 5% CO_2_.

### Transfection assay

We obtained the overexpression plasmid of JAM3 and an empty plasmid from GeneCopoeia (Guangzhou, China). Firstly, we planted BC cells into 6-well plates and waited for cell density to reach 80%–90%. Then, we changed the complete culture medium into 1.5-mL Opti-MEM (Gibco) for each well, 2 h before transfection. Two groups were set up for transfection: the negative control (NC) group and the overexpression (OE) group. For the OE group, 10 uL of the JAM3 overexpression plasmid was added to 240 uL of Opti-MEM for each well. To this, 5 uL of lipo2000 (Thermo, USA) was added to 245 uL of Opti-MEM and allowed to sit at room temperature for 5 min. The plasmid and lipo2000 solutions were then mixed and allowed to sit for an additional 10 min. The resulting transfection solution (500 ul) was added to each well, resulting in a 2-mL volume transfection system. The NC group followed the same protocol; however, the overexpression plasmid was replaced with an empty plasmid. After 8 h of transfection, the culture medium was changed to 2 mL of RPMI-1640 supplemented with 10% serum (DMEM was used as the basic medium for UMUC3 cells). Transfection efficacy was confirmed by examining the fluorescence percentage and wells with a minimum of 80% fluorescence were selected for further investigation.

### Wound healing assay

We planted OE cells and NC cells into 6-well plates and allowed them to reach full cell density. Then, we utilized a 200-uL pipette tip to create lines and generate wounds. After washing with a PBS solution three times, we supplemented each well with 2 mL of RPMI-1640 basic medium (DMEM for UMUC3 cells) and captured images at 0, 24, and 48 h for analysis of the wound healing assay results.

### Transwell assay

This section was divided into migration and invasion assay. For migration assay, the transinfected cells were incubated in a serum-free medium for 12 h and then adjusted to a density of 1×10^6^ cells/mL. Next, 200-µL serum-free cell suspension was added to the transwell chambers (Corning, No.3422), which were fit into the wells of 24-well plates. The wells of the plates also each contained 500 µL of basic medium supplemented with 10% FBS. After 24 h of incubation, cells on the upper membrane were removed with cotton wool, whereas cells adhering to the lower surface were fixed in methanol for 30 min and then stained with 0.1% crystal violet for 20 min. After natural air drying, migrating cells on the lower surface of the membrane were then counted under an optical microscope at 200× magnification. For invasion assay, the same protocol was followed except that the transwell chambers were pretreated with Matrigel extracellular matrices (Corning, USA). The dilution ratio for the extracellular matrices was 1:8.

### Western blot assay

Before we processed the western blot assay, we used a BCA protein assay kit (Beyotime, Beijing) to determine the loading sample. Twenty micrograms of total protein were separated by SDS-PAGE on 12% gradient polyacrylamide gels. Gels were electroblotted onto nitrocellulose membranes. For immunodetection, blots were blocked with 1% blocking reagent in 0.05% Tween 20-PBS for 1 h and incubated with primary antibody overnight at 4 ^∘^C diluted in blocking buffer. The dilutions used in Western blots were anti-beta-Tublin (1:5000), anti-N-Cadherin (1:2000), anti-Claudin-1 (1:1000), anti-matrix metallopeptidase 2 (MMP2) (1:1000), and anti-JAM3 (1:5000). The anti-JAM3 was purchased from Abmart (Shanghai, China), while the remaining primary antibodies were purchased from Abcam. Blots were then washed in 0.05% Tween 20-PBS and incubated with goat anti-rabbit (1:25000) (Abclonal, China) peroxidase-labeled antibody in a blocking buffer for 1 h. An enhanced chemo luminescent system was applied. Scanning densitometry was performed with scan analysis software.

### Ethical statement

TCGA database and GEO database are public databases, and there is no ethical conflict. Meanwhile, no ethics statement was required from the institutional review board for the use of these prostate cancer cell lines.

### Statistical analysis

We employed one-way ANOVA and *t* test for comparison between groups, and the comparison of two or more constituent ratios was used by the chi-square test. Correlation analysis, heatmaps, receiver operating characteristic (ROC) curves, box plots, and violin plots were completed by R software (version 4.2.1). Data of pan-cancers was downloaded from the TIMER 2.0 database (http://timer.comp-genomics.org/). Statistics of relative protein expressions were completed by using ImageJ and Graphpad Prism software (version 10.0.2). R software was used for additional statistical analyses. A significance level of *P* < 0.05 was deemed appropriate for this study.

## Results

### 172 differentially expressed genes (DEGs) were screened from the vast GEO cohort

At first, we obtained a vast GEO cohort containing the expression of 9972 genes by merging six GEO datasets (GSE3167, GSE13507, GSE52519, GSE65635, GSE100926, and GSE120736), which included 87 normal samples as controls and 394 BC samples. Using an FDR threshold of less than 0.05 and a |log2 (FC)| of at least 1, we identified 172 DEGs (24 upregulated and 148 downregulated) between the BC samples and controls. All DEGs are shown by the volcano map, and the most relevant 100 DEGs are depicted in the heatmap (Figure S1A and S1B). Our GO and KEGG enrichment analysis revealed that these DEGs are primarily associated with extracellular matrix (ECM) functions and pathways (Figure S1C and S1D) (qvalue < 0.05). Furthermore, our DO analysis highlighted a significant enrichment in urinary system cancer (qvalue < 0.05), further confirming the strong connection between these DEGs and BC (Figure S1E).

Furthermore, we conducted GSEA to investigate the pathways enriched in the normal and BC tissues. We found that pathways related to cell adhesion molecules, cytokine–cytokine receptor interaction, and focal adhesion were active in normal tissues. In contrast, pathways commonly associated with tumor development, such as cell cycle, DNA replication, and BC, were predominantly active in BC tissues (Figure S1F and S1G). These findings preliminarily showed the strong relationship between DEGs and BC.

### 58 BC-related genes were obtained by applying weighted correlation network analysis (WGCNA)

**Figure 1.**
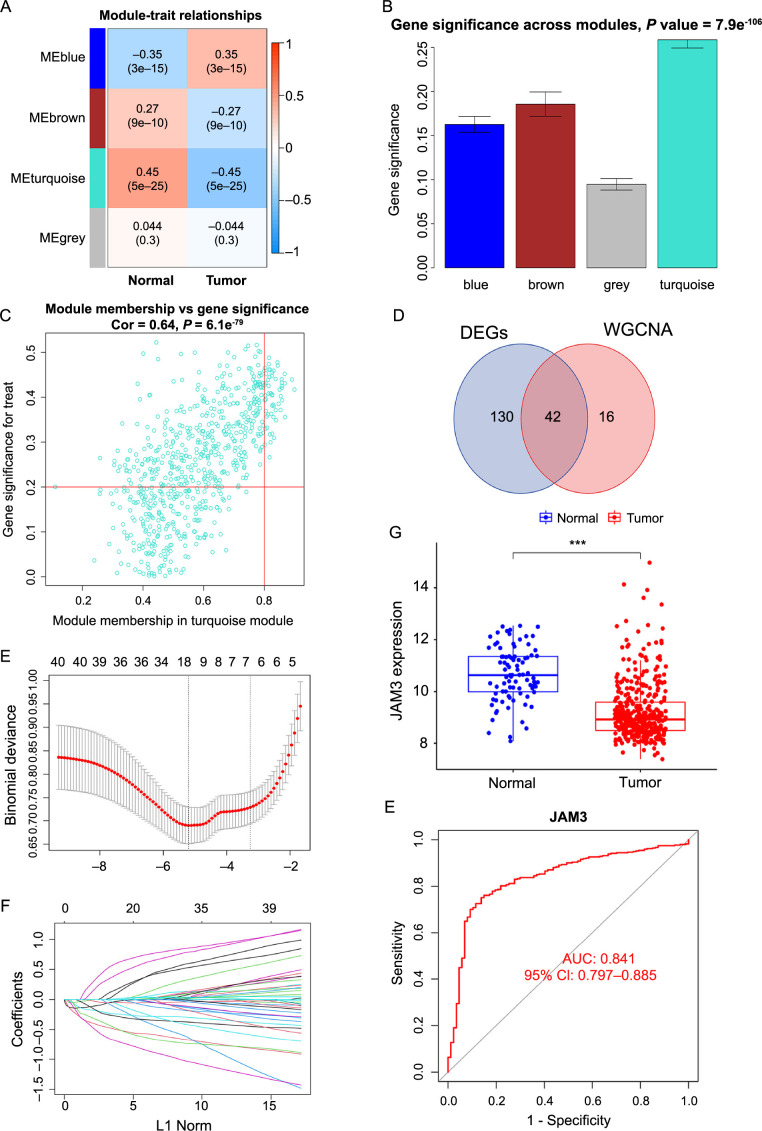
**Construction of BC-related gene co-expression modules and gene screening.** (A) Heatmap of the correlation between module eigengenes and clinical traits of BC; (B) Distribution of average gene significance and errors in the modules associated with BC; (C) Scatter plot of module eigengenes related to BC in the turquoise module; (D) Venn plot showing 42 overlapping genes between 172 DEGs and 58 genes which filter from WGCNA; (E) Tenfold cross-validation for the 42 overlapping genes in the LASSO analysis; (F) LASSO coefficient profiles of 42 overlapping genes for BC; (G) Boxplot of JAM3 expression across BC and normal samples in GEO cohort; (H) ROC curve of JAM3 as a diagnostic gene for BC in GEO cohort. ***P < 0.001. BC: Bladder cancer; DEG: Differentially expressed genes; WGCNA: Weighted correlation network analysis; LASSO: Least absolute shrinkage and selection operator; JAM3: Junctional adhesion molecule 3; GEO: Gene Expression Omnibus; ROC: Receiver operating characteristic.

In order to explore the genes with similar expression patterns among the 9972 genes of BC, we adopted the WGCNA analysis to construct the gene co-expression network. First, we evaluated the sample clustering dendrogram of 481 samples (Figure S2A). Next, we established the ideal soft thresholding power which mainly influenced the scale independence and mean connectivity of gene co-expression modules. We chose seven as the power value based on the analysis of the scale-free index and mean connectivity for various soft-threshold powers (Figure S2B), as well as the selected power value, could construct a scale-free network well (*R*^2^ ═ 0.88) (Figure S2C). Subsequently, these 9972 genes were divided into four different co-expression modules, represented by different colors (Figure S2D and S2E). Additionally, we created a heatmap to illustrate the correlation analysis findings between each module and clinical traits (Figure 1A), and we discovered that the turquoise module had the most significant (*r* ═ −0.45, *P* ═ 5e-25) association with the BC. Similarly, the turquoise module was the most important in determining whether the sample belonged to BC or not (Figure 1B). Finally, we obtained 58 related genes from the turquoise module when we set “module membership” to 0.8 and “gene significance” to 0.2 (Figure 1C). In summary, we obtained the 58 related genes most closely associated with BC by constructing a gene co-expression network.

### JAM3 was an independent prognostic factor associated with poor prognosis in BC

At first, we intersected the 172 DEGs with the 58 related genes from the previous step, and the Venn diagram showed 42 overlapping genes (Figure 1D). Then, the 42 intersected genes were analyzed by LASSO regression analysis and finally 16 genes (*HSPB6*, *DIXDC1*, *CNN1*, *SPARCL1*, *DCN*, *FLNC*, *FHL1*, *BIN1*, *JAM3*, *RASL12*, *PDLIM3*, *FXYD6*, *PLA2G4C*, *TGFB3*, *COL6A2*, and *PTRF*) were identified (Figure 1E and 1F). On the one hand, it was clear that all 16 genes had considerably lower expression levels in BC tissues than in normal tissues in our GEO cohort, on the other hand, the accuracy of these genes as diagnostic genes was quite high, with all AUC values of more than 0.79, including JAM3 (Figure S1G and S1H, Supplementary material 1 and 2). A similar trend was observed in the TCGA cohort, except for gene *PTRF*. The remaining 15 genes also exhibited lower expression in BC with high diagnostic values accuracy (Figure S2F and S2G, Supplementary material 3 and 4).

**Figure 2.**
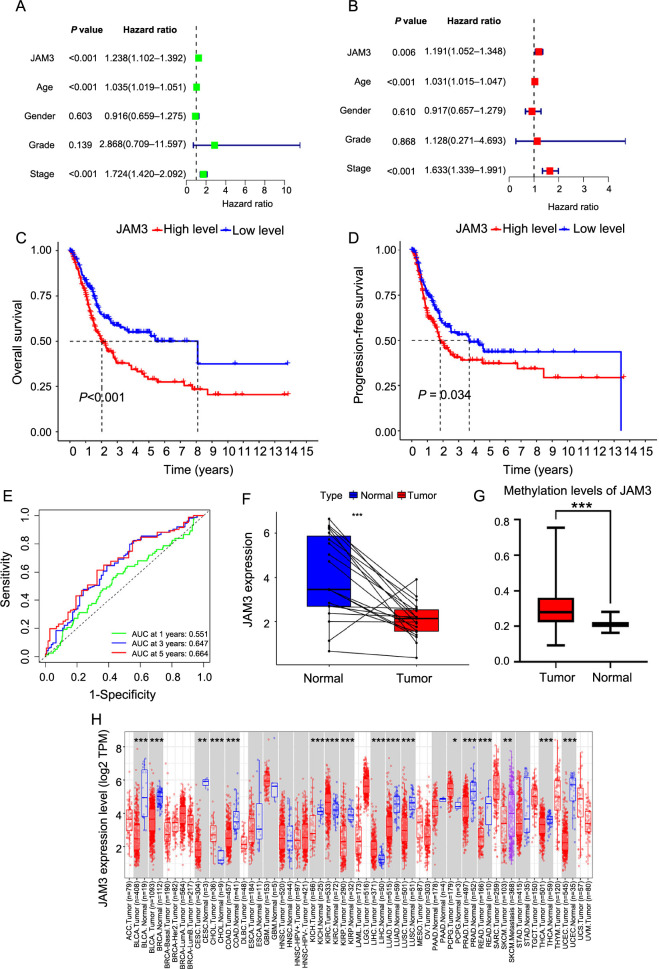
**Association of JAM3 with prognosis of BC.** (A and B) Univariate and multivariate Cox regression analysis; (C and D) Kaplan–Meier analysis for OS and PFS of JAM3 in BC; (E) Time-dependent ROC curves and AUC at 1-, 3-, and 5-year were used to evaluate the predictive value of JAM3; (F) Paired expression analyses of JAM3 in TCGA cohort for BC; (G) Difference in M6A methylation level of JAM3 between BC and normal samples; (H) The expression of JAM3 in pan-cancer. *P < 0.05, **P < 0.01 ***P < 0.001. OS: Overall survival; PFS: Progression-free survival; JAM3: Junctional adhesion molecule 3; TCGA: The Cancer Genome Atlas; ROC: Receiver operating characteristic; AUC: Area under the curve; JAM3: Junctional adhesion molecule 3; BC: Bladder cancer.

To further investigate the effect of these 15 genes on the prognosis, we performed univariate and multivariate cox regression analysis on BC samples from the TCGA cohort by using the survival package of R. From the results, we found a close correlation between JAM3, age, and stage and the survival of patients (Figure 2A and 2B). Besides, JAM3 was the only independent prognostic factor among the 15 genes that acted as a risk factor in BC. Substantially, we divided these BC samples into two groups based on the median of JAM3 expression: the high-expression (HE) group and the low-expression (LE) group.

**Figure 3.**
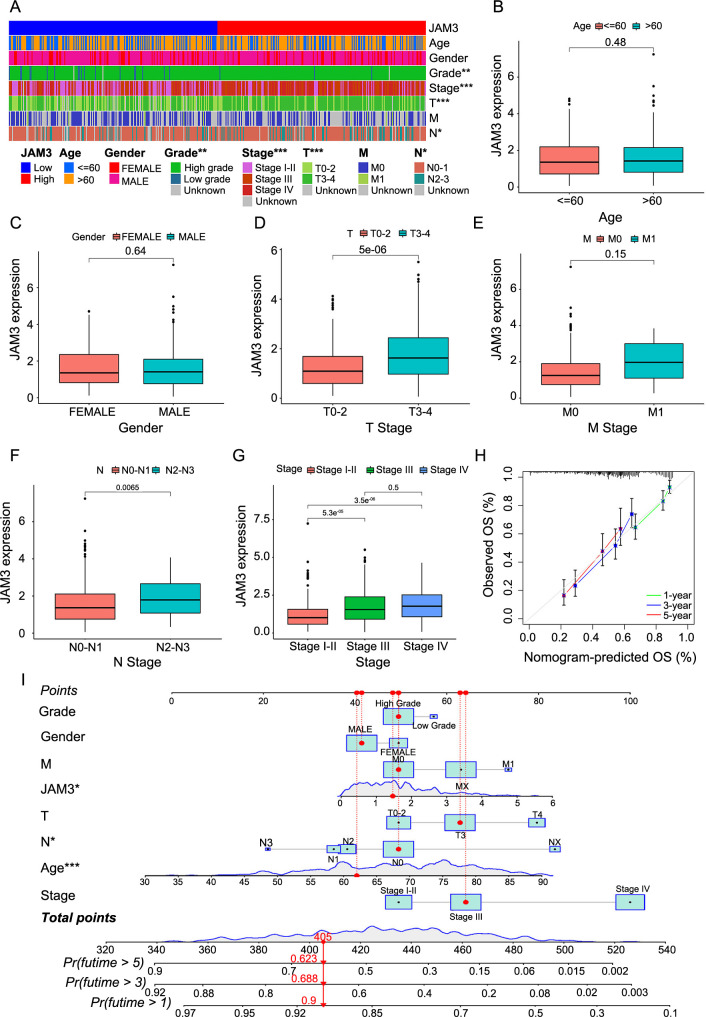
**Relationship between JAM3 expression level and clinical parameters.** Difference analysis of (A) Clinical traits in high and low JAM3 expression groups, and (B–G) JAM3 expression in different clinical traits; (H) 1-, 3-, 5-year calibration diagrams describing nomogram performance; (I) Nomogram was established to predict the risk score and survival probability of BC patients. ***P < 0.001. JAM3: Junctional adhesion molecule 3; OS: Overall survival; Pr: Probability.

Next, we conducted a Kaplan–Meier analysis to assess the overall survival (OS) and progression-free survival (PFS). The Kaplan–Meier curve suggested that the HE group had a much lower survival rate than the LE group (Figure 2C and 2D). Meanwhile, ROC curves showed that JAM3 was effective in predicting 1-, 3-, and 5-year survival rates, which were 0.551, 0.647, and 0.664, respectively (Figure 2E). Survival analysis of external datasets (GSE13507, GSE48276, and GSE37817) also supported our results (Figure S5A). Overall, JAM3 was strongly associated with poor prognosis in BC. Interestingly, JAM3 levels were lower in tumors compared to paired samples from the TCGA cohort in BC (Figure 2F). We also observed a higher methylation level of JAM3 in BC, which aligns with previous studies and suggests that methylation may influence JAM3’s functions (Figure 2G). Additionally, using the website TIMER 2.0, we found that JAM3 had higher expression in five types of tumors and lower expression in 12 types of tumors, including BC (Figure 2H). These findings suggest that JAM3 is a promising prognostic gene that warrants further investigation.

### BC patients with high levels of JAM3 exhibited worse clinical parameters

To better understand the significance of JAM3 in a clinical setting, we created a heatmap (Figure 3A) that visually represents the correlation between JAM3 expression levels and key clinical parameters, such as age, gender, grade, stage, T stage, N stage, and M stage. Additional clinical information can be found in Supplemental sheet 1 and Table S1. Our analysis revealed significant differences between the HE and LE groups, particularly in regards to tumor grade (categorized as high or low), stage (categorized as I-II, III, or IV), T stage (categorized as T0-2 or T3-4), and N stage (categorized as N0-1 or N2-3). These findings demonstrate that high levels of JAM3 are closely linked to unfavorable clinical outcomes, including higher tumor grade, T and N stages, and overall tumor stage (Figure 3B–3G, Figure S6). These results were confirmed by an external dataset, the GEO dataset (GSE13507, GSE48276, and GSE37817), which further supports the association between JAM3 expression and clinical parameters. Furthermore, our analysis also showed a significant increase in JAM3 expression in MIBC (Figure S5B–S5H), suggesting that JAM3 expression can be an effective indicator of clinical parameters.

We developed a prognostic nomogram by merging clinical features and JAM3 to evaluate the usefulness of applying JAM3 in predicting BC prognosis (Figure 3I). We also generated calibration plots for 1-, 3-, and 5-year predictions to assess the accuracy of our nomogram model, which demonstrated its capability to accurately forecast survival (Figure 3H). In conclusion, these findings suggest that JAM3 holds promising potential for clinical use.

### Biofunctional analysis indicated JAM3 was related to calcium signaling pathway and PI3K-Akt signaling pathway, as well as the EMT-related proteins

**Figure 4.**
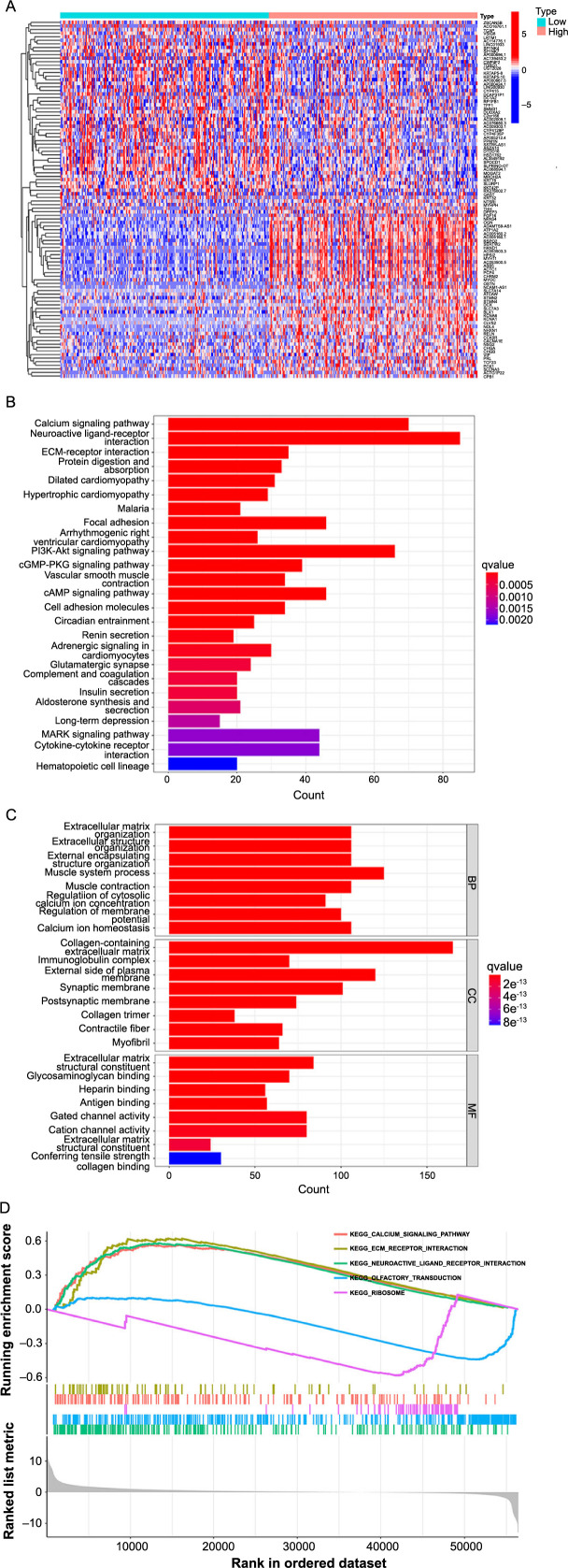
**Enrichment analysis of the differential genes between HE and LE groups**. (A) Heatmap showing the 100 genes with the most significant differences; (B and C) GO analysis and KEGG analysis revealed potential biological functions and pathways involved in JAM3; (D) GSEA analysis showing the active pathways in HE and LE groups. HE: High-expression group; LE: Low-expression group; KEGG: Kyoto Encyclopedia of Genes and Genomes; GO: Gene ontology; JAM3: Junctional adhesion molecule 3; GSEA: Gene Set Enrichment Analysis.

Additionally, our analysis revealed a total of 2565 DEGs when using a threshold of FDR < 0.05 and |log2(FC)|≥1 to compare the HE group and LE group. Figure 4A shows a heatmap featuring the 50 most significantly upregulated and downregulated genes.

We utilized JAM3-related DEGs to conduct the GO function and KEGG pathway enrichment analysis (qvalue < 0.05). Our analysis revealed that these DEGs were enriched in the calcium signaling pathway, neuroactive ligand–receptor interaction, ECM–receptor interaction, and the PI3K-Akt signaling pathway (Figure 4B). GO enrichment analysis showed that the most significant functions were related to the ECM (Figure 4C). Additionally, our GSEA analysis demonstrated that the HE group showed activity in the calcium signaling pathway, ECM-receptor interaction, and neuroactive ligand–receptor interaction (Figure 4D).

We observed that the JAM3 levels have an impact on the expression of various EMT-related genes, according to a comparison between the HE and LE groups. Within these genes, *ZEB1*, *CDH2*, *MMP2*, *VIM*, and *SNAI1* were significantly upregulated by over 1 logFC (with a *P* value of less than 0.05) in the HE group. This leads us to believe that elevated levels of JAM3 may regulate EMT-related proteins, providing a potential explanation for the prognostic role of JAM3.

### JAM3 overexpression enhances migration and invasion capabilities in BC cells through the EMT process regulation

**Figure 5.**
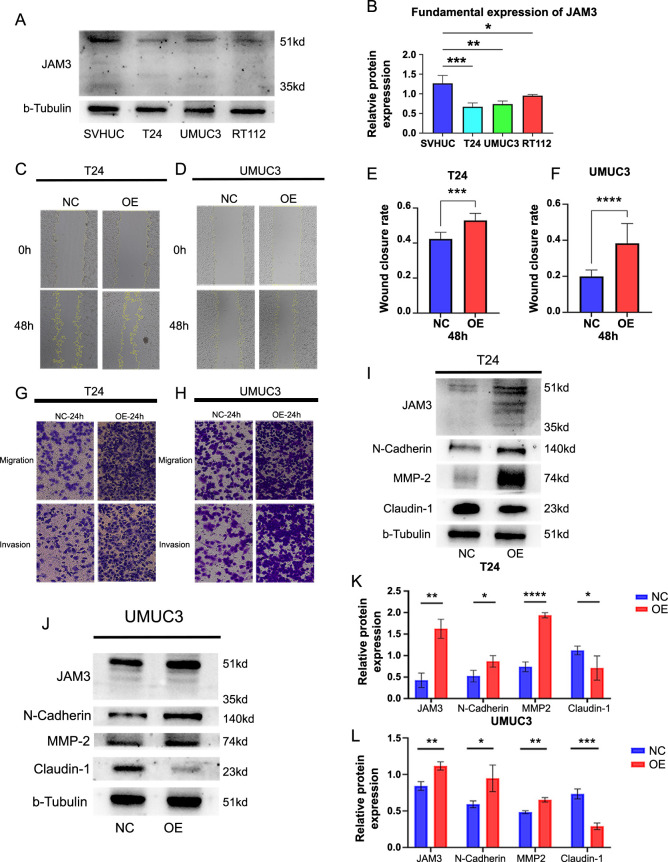
**Overexpressed JAM3 promoted BC cell migration and invasion abilities by regulating expression levels of EMT-related genes**. (A and B) The fundamental expression of JAM3 in normal bladder cells and BC cells; (C and D) Wound heling assay for overexpressed T24 cells (C) and UMUC3 cells (D); (E and F) Statistical results of wound healing assay for T24 cells (E) and UMUC3 cells (F); (G and H) Migration and invasion assay for overexpressed T24 cells (G) and UMUC3 cells (H); (I and J) Overexpressed JAM3 upregulated levels of N-Cadherin and MMP-2 proteins, while inhibited the level of Claudin-1 protein in T24 cells (I) and UMUC3 cells (J); (K and L) The relative protein expression level of EMT-related proteins, calculation followed protocol as below: gray levels of targeted proteins divided by gray levels of paired tubulin protein. *P < 0.05, **P < 0.01, ***P < 0.001, ****P < 0.0001. NC: Negative control group; OE: Overexpressed group; JAM3: Junctional adhesion molecule 3; UMUC3: Bladder cancer cell line; EMT: Epithelial–mesenchymal transition; BC: Bladder cancer.

To verify our speculation, we conducted a series of experiments. Firstly, we analyzed the fundamental expression of JAM3 in human normal bladder cells and BC cells. The results showed that JAM3 expression in BC cell lines T24, UMUC3, and RT112 was lower compared to SVHUC cells, consistent with our previous bioinformatic analysis (Figure 5A and 5B). To further investigate, we overexpressed JAM3 in T24 and UMUC3 cells using a plasmid. After confirming at least 80% fluorescence, we assessed the migration and invasion abilities of the cells through transwell assays. We observed higher wound healing rates (Figure 5C–5F) and increased migration and invasion abilities within 24 h (Figure 5G and 5H) in the overexpressed cells. To determine the impact of JAM3 overexpression on EMT-related proteins, we conducted a western blot assay on cell proteins extracted after 48 h of transfection. We found significantly higher levels of JAM3 in the OE group compared to the NC group in T24 and UMUC3 cells, as well as increased levels of N-Cadherin and MMP-2 proteins and decreased levels of Claudin-1 (Figure 5I–5L). These results suggest that overexpressed BC cells acquire characteristics of mesenchymal cells, promoting EMT and providing initial support for our speculation.

### JAM3 was related to poor immune functions in BC

**Figure 6.**
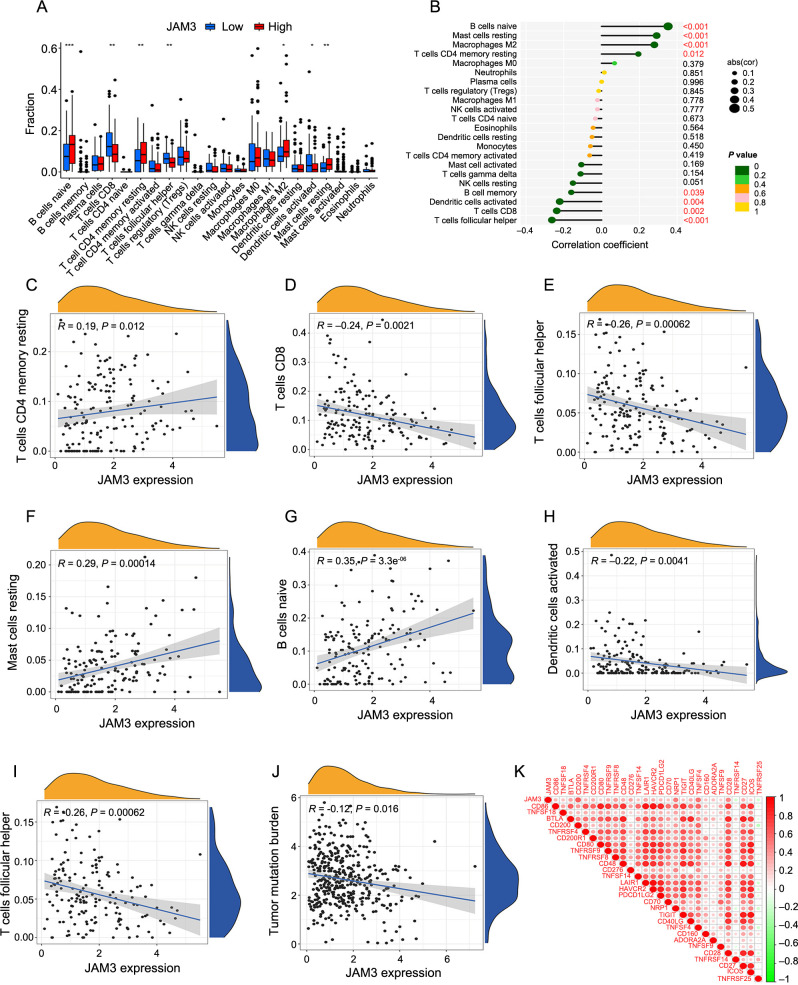
**Correlation analysis between JAM3 and tumor-infiltrating immune cells**. (A) The difference of 22 immune cell infiltration between HE and LE groups; (B) Relationships between the expression of JAM3 and 22 types of tumor-infiltrating immune cells; (C–I) Correlation of JAM3 expression with naive B cells, resting memory CD4 T cells, M2 macrophages, resting mast cells CD8 T cells, follicular helper T cells and activated dendritic cells; (J) Relationship between JAM3 expression and tumor mutation burden; (K) Correlation between JAM3 and immune checkpoints. **P < 0.01, ***P < 0.001, ****P < 0.0001. JAM3: Junctional adhesion molecule 3; HE: High-expression group; LE: Low-expression group; abs: Absolute value.

Using the CIBERSORT algorithm, we investigated the connection between JAM3 and tumor immunity by calculating the infiltration of 22 different immune cell types in each BC sample. Analysis of JAM3 expression levels showed higher infiltration degrees of naive B cells, resting memory CD4 T cells, M2 macrophages, and resting mast cells in the HE group, while CD8+ T cells, follicular helper T cells, and activated dendritic cells showed higher levels in the LE group (Figure 6A). Interestingly, the four immune cell types with higher infiltration in the HE group were significantly positively correlated with JAM3 expression, while memory B cells and the three immune cell types with higher infiltration in the LE group showed a significant negative correlation with JAM3 expression (Figure 6B). Figure 6C–6I provides scatter plots for more a detailed visualization of the correlation. These findings suggest that JAM3 is associated with poor immune cell infiltration in BC, highlighting its relevance to poor patient prognosis.

In our study, we observed a negative correlation (*R* ═ −0.12) between tumor mutation burden and JAM3 expression (Figure 6J). We also noted that JAM3 exhibited a positive correlation with immune checkpoints CD200, NRP1, TNFSF4, and CD28, with notably high values greater than 0.4 (Figure 6K). These findings suggest that JAM3 could potentially serve as a predictor for the efficacy of immunotherapy.

### Co-expression network of JAM3 in BC

We utilized the String website to construct a PPI network in order to investigate the capabilities of JAM3 (Figure S3A). A total of 275 genes showed a strong correlation with JAM3 (|r|>0.6) (Supplementary material 5). The diagram (Figure S3B) displays the top 11 genes that are highly correlated with JAM3, including *S100A11, NT5C, SYTL1, STXBP2, AC068831.5* (negatively correlated with JAM3), and *DCHS1, ADGRA2, ZEB1, MAP1A, PRKG1, and ZNF521* (positively correlated with JAM3). Additional information can be found in the correlation diagrams (Figure S3C). Previous studies have shown that among these genes, *MAP1A, ZEB1,* and *ZNF521* are associated with poor behavior or prognosis in BC [29–31]. These findings will be valuable as we further examine the role of JAM3.

## Discussion

The most common cancer in the urinary system is BC, which has been shown to have various characteristics. Changes in DNA and RNA may contribute to its clinical and pathological features [32, 33]. In our investigation, we found JAM3 to be an independent prognostic factor for BC, although its role in cancer is debated. It has been identified as an oncogene in renal cell and small cell lung carcinoma but as a suppressor in colorectal and esophageal cancer [24, 25, 34, 35]. In previous studies on BC, a multiple-gene model was constructed to predict patient prognosis, although the specific role of JAM3 as a risk factor was not thoroughly explored [36]. Our findings demonstrate the value of high levels of JAM3 as a prognostic factor for predicting a poor prognosis, worse clinical outcomes, and decreased immune cell infiltration in BC.

In our study, we observed that BC cells with elevated levels of JAM3 displayed enhanced migration and invasion abilities, as demonstrated by the transwell assay. Furthermore, our results indicate that overexpression of JAM3 resulted in increased expression of N-Cadherin and MMP2 proteins, while Claudin-1 protein levels were decreased. The increasing level of N-Cadherin was considered a landmark of epithelial feature loss and the gain of mesenchymal features, which is usually accompanied by decreased E-Cadherin level and promoted the tumor invasion abilities [37]. Besides, the cadherin proteins were closely related to calcium ions, which was consistent with our bioinformatic findings. Additionally, MMP2 upregulation can facilitate tumor cell invasion by breaking down the ECM [27], while Claudin-1 downregulation can disrupt cell–cell adhesion during the process of EMT [38]. Similarly, JAM3 was found highly expressed in renal carcinoma cells, inhibited tumor cell apoptosis, and promoted cell migration by upregulating levels of N-Cadherin, integrin β1, and MMP-2 [34]. These findings distinguished that a high level of JAM3 promoted cell migration and invasion by regulating the EMT process in BC, eventually leading to tumor progression and metastasis. However, further verification is required as western blot analysis lacks quantitative data.

Importantly, the results of GO, KEGG, and GSEA analysis showed that these JAM3-related DEGs were mainly concentrated in functions and pathways associated with the calcium signaling pathway, ECM, and PI3K-Akt signaling pathway. It has been demonstrated that calcium ion channels and pumps are abnormally expressed in various tumors, with a complex influence on tumor cell proliferation, metastasis, invasion, and drug resistance [39]. Also, it is reported that high serum calcium level was proved to be an independent risk factor for bone metastases in BC patients, highlighting the important functions of calcium ions in BC [36]. ECM of tumor plays a fundamental and dynamic role in the development of the TME, the growth of tumor cells is often supported by widespread biochemical and biomechanical alterations in the tumor matrix [40, 41]. Besides, ECM also tends to support the tumor’s proliferation and migration, and suppresses anti-tumor immune function in BC [42, 43]. In tumor cells the PI3K-AKT pathway is often overactivated, and the aberrant activation of this pathway is closely related to the occurrence and development of a variety of tumors [44–46], thus becoming an important target in cancer research. The PI3K-AKT pathway plays a key regulatory role in tumor cells, and participates in a number of biological processes, such as cell survival, proliferation, apoptosis, and metabolism. And the results of enrichment analysis also provide clues that the role of JAM3 in BC cells, such as promoting EMT, may be accomplished through the PI3K-AKT pathway. This also refers to the next study direction: in BC, JAM3 promotes tumor growth via the PI3K-AKT pathway, including proliferation, migration, and invasion. However, since these analyses are based on public databases, which have certain shortcomings, such as the variability among individual patients, the results may be biased. The precise role of JAM3 in BC is still undetermined. Therefore, it is reasonable to speculate that the risk role of JAM3 in BC is closely related to calcium signaling, the ECM, and the PI3K-Akt signaling pathway, which could be the direction for further investigation. Our aim is to confirm this hypothesis through the use of clinical samples in future studies.

TME plays an important role in tumor initiation, progression, invasion, and spread [47]. Several articles have provided constructive insights into the development and treatment of BC based on the immune genes and the TME [48–50], and the existence of a close association between JAM3 and a variety of immune cells suggests to us that there exists a certain correlation between JAM3 and the immune microenvironment of BC, which we believe can be further elucidated in the future studies.

Consistently, we found that a high level of JAM3 was positively associated with infiltration levels of resting mast cells and M2 macrophages. M2 macrophages promote tumor angiogenesis and tumor cell development, blocking the function of T cells, and are associated with poor prognosis, also in BC [51, 52]. While, mast cells exert pro- or anti-tumor effects due to different tumors, including BC and different TMEs [53, 54]. Meanwhile, a significant negative correlation with activated dendritic cells and CD8+ T cells was observed as well. Activation of CD8+ T cells was crucial in tumor immunity, but a key problem with tumor antigen presentation for effective antitumor response is dendritic cells must effectively take up and present tumor antigens and subsequently activate CD8 + T cells [55]. In addition, JAM3 was associated with the CD200, an inhibitory immune checkpoint [56] that suppressed anti-tumor immune function by binding its receptor CD200R on myeloid cells [57, 58]. These immunological aspects of the analysis above corroborate to some extent the association of JAM3 with the TME in BC.

There are undoubtedly some limitations in our research, including differences in ethnicity within the GEO and TCGA datasets, a limited sample size, and a lack of in-depth mechanistic analysis. As a result, further investigation is needed to clarify the role of JAM3 in BC.

## Conclusion

In conclusion, we identified a high level of JAM3 as a valuable independent factor for predicting poor prognosis by regulating the EMT process, as well as predicting the bad immune infiltrations, which provides a new biomarker for determining the prognosis and immune functions of BC patients.

## Supplemental data

Supplementary figures and table: https://www.bjbms.org/ojs/index.php/bjbms/article/view/9979/3166

Supplementary material 1: https://www.bjbms.org/ojs/index.php/bjbms/article/view/9979/3229

Supplementary material 2: https://www.bjbms.org/ojs/index.php/bjbms/article/view/9979/3230

Supplementary material 3: https://www.bjbms.org/ojs/index.php/bjbms/article/view/9979/3231

Supplementary material 4: https://www.bjbms.org/ojs/index.php/bjbms/article/view/9979/3232

Supplementary material 5: https://www.bjbms.org/ojs/index.php/bjbms/article/view/9979/3233

Supplementary sheet: https://www.bjbms.org/ojs/index.php/bjbms/article/view/9979/3165

## Data Availability

The data generated in this study are publicly available in Gene Expression Omnibus (GEO) at GSE3167, GSE13507, GSE52519, GSE65635, GSE100926 and GSE120736, and in The Cancer Genome Atlas (TCGA) for bladder cancer data.
